# Differential Thermoregulatory and Inflammatory Patterns in the Circadian Response to LPS-Induced Septic Shock

**DOI:** 10.3389/fcimb.2020.00100

**Published:** 2020-03-12

**Authors:** Malena Lis Mul Fedele, Ignacio Aiello, Carlos Sebastián Caldart, Diego Andrés Golombek, Luciano Marpegan, Natalia Paladino

**Affiliations:** Laboratorio de Cronobiología, Departamento de Ciencia y Tecnología, Universidad Nacional de Quilmes/CONICET, Buenos Aires, Argentina

**Keywords:** sepsis, circadian rhythms, immune system, Tumor Necrosis Factor-α, hypothermia

## Abstract

Sepsis is caused by a dysregulated host response to infection, and characterized by uncontrolled inflammation together with immunosuppression, impaired innate immune functions of phagocytes and complement activation. Septic patients develop fever or hypothermia, being the last one characteristic of severe cases. Both lipopolysaccharide (LPS) and Tumor Necrosis Factor (TNF)-α- induced septic shock in mice is dependent on the time of administration. In this study, we aimed to further characterize the circadian response to high doses of LPS. First, we found that mice injected with LPS at ZT11 developed a higher hypothermia than those inoculated at ZT19. This response was accompanied by higher neuronal activation of the preoptic, suprachiasmatic, and paraventricular nuclei of the hypothalamus. However, LPS-induced *Tnf-*α and *Tnf-*α type 1 receptor (TNFR1) expression in the preoptic area was time-independent. We also analyzed peritoneal and spleen macrophages, and observed an exacerbated response after ZT11 stimulation. The serum of mice inoculated with LPS at ZT11 induced deeper hypothermia in naïve animals than the one coming from ZT19-inoculated mice, related to higher TNF-α serum levels during the day. We also analyzed the response in TNFR1-deficient mice, and found that both the daily difference in the mortality rate, the hypothermic response and neuronal activation were lost. Moreover, mice subjected to circadian desynchronization showed no differences in the mortality rate throughout the day, and developed lower minimum temperatures than mice under light-dark conditions. Also, those injected at ZT11 showed increased levels of TNF-α in serum compared to standard light conditions. These results suggest a circadian dependency of the central thermoregulatory and peripheral inflammatory response to septic-shock, with TNF-α playing a central role in this circadian response.

## Introduction

Sepsis is a syndrome characterized by a dysregulated host response to a pathogen and is the primary cause of death from infection (Singer et al., 2016). In the United States, the incidence of severe sepsis is more than 3 per 1,000 persons (Kumar et al., 2011) and the in-hospital mortality rate is about 25–30% of septic patients worldwide (Vincent et al., 2014). Therefore, it is important to recognize early signs to treat this syndrome and to avoid its progression, as there is still no effective treatment available. Sepsis symptoms include: body temperature alterations (fever or hypothermia), elevated heart and respiratory rate, hyperglycemia, alterations of inflammatory and hemodynamic variables, among others (Angus and van der Poll, 2013). Septic shock is defined as the septic condition worsened by metabolic and circulatory alterations, as hypotension, which increase the mortality rate (Singer et al., 2016).

As it is a complex and multi-symptomatic pathology, it may be important to differentiate between those symptoms related with more severe cases from those compatible with a better prognosis. For example, it has been shown that hypothermia is related to a worse prognosis than fever (Remick and Xioa, 2006; Rumbus et al., 2017). Body temperature is controlled by neuronal circuits present, mainly, in the hypothalamus (Morrison, 2016). Particularly, the hypothalamic preoptic area (POA) is the main integrative brain site for thermoregulation, controlling brain and peripheral body temperature (Boulant, 1998; Morrison and Nakamura, 2018).

Pathogen infection also triggers an important inflammatory response. The innate immune response induces an increase of pro-inflammatory cytokine release known as “cytokine storm” (Bosmann and Ward, 2013). On the other hand, an anti-inflammatory response, including glucocorticoid secretion (Marik, 2011), is also elicited. The pro-inflammatory mechanisms contribute to clear the infection while the anti-inflammatory ones respond to tissue healing. However, an excess of inflammation can cause tissue damage while an excess of anti-inflammatory responses can produce secondary infections (van der Poll and Opal, 2008).

One of the main cytokines secreted in response to sepsis is Tumor Necrosis Factor (TNF)-α. In animal models, TNF-α administration induces most of septic symptoms and signs (Tracey et al., 1988). Moreover, lack of TNF-α signaling, as in TNF-α receptor (TNFR) deficient (KO) mice or generated by soluble TNFR administration, induces higher resistance to sepsis (Tracey et al., 1987; Mohler et al., 1993; Pfeffer et al., 1993; Guo et al., 2009).

One of the most studied animal models of sepsis is induced by the administration of high doses (close to 20 mg/kg) of the bacterial endotoxin lipopolysaccharide (LPS), producing high mortality rates (Li et al., 2014; Liao and Lin, 2015; Ramos-Benitez et al., 2018) and the characteristic signs of sepsis: pro-inflammatory cytokine induction (Ogawa et al., 2016; Ramos-Benitez et al., 2018), hypothermia (Saito et al., 2003; Nautiyal et al., 2009), hypotension (Chuaiphichai et al., 2016) and immunosuppressive response development (Córdoba-Moreno et al., 2018), among others.

In addition, there is a daily variation in the mortality rate due to septic shock: mice intraperitoneally injected with high doses of LPS at the end of the day show a higher mortality rate than those injected in the middle of the night (Halberg et al., 1960; Marpegan et al., 2009). Similar results were obtained when TNF-α was administered intravenously (Hrushesky et al., 1994). These experiments indicated that the response triggered by sepsis is related to the circadian system, linked to a central biological clock in the hypothalamic suprachiasmatic nuclei (SCN) which is mainly synchronized by the light-dark (LD) cycle (Golombek and Rosenstein, 2010). Moreover, mice deficient for the clock genes Period 2 (PER2) or Clock are more resistant to septic shock (Liu et al., 2006b; Wang et al., 2016). Finally, several studies observed that animals subjected to circadian desynchronization, such as SCN lesions or experimental protocols of chronic jet-lag (CJL), exhibit an increase in the inflammatory response triggered after LPS administration (Castanon-Cervantes et al., 2010; Adams et al., 2013; Guerrero-Vargas et al., 2014).

Despite the numerous evidences that show a circadian component of the septic response, the causes that generate this differential response are still unknown. The aim of this study was to further analyze the interaction between the circadian system and the mechanisms triggered during sepsis, to better understand and differentiate lethal and survival responses.

## Materials and Methods

### Animals

Adult (2-month old) C57BL/6J wild type (WT) and TNFR1-deficient (TNFR1 KO) male mice (*Mus musculus*) were raised in our colony. TNFR1 KO mice (originally from The Jackson Laboratory—B6.129-Tnfrsf1a^tm1Mak^/J—raised in a C57BL/6J background) were kindly provided by Dr. Silvia Di Genaro (San Luis National University, Argentina). The neomycin cassette present in KO mice in the position 535 of the coding sequence of Tnfr1 was detected by polymerase chain reaction (PCR) following the instructions described by The Jackson Laboratory (data not shown). All mice were housed in groups under a 12:12-h LD photoperiod (with lights on at 7 a.m. and lights off at 7 p.m.) with food and water *ad libitum*. This study was carried out in accordance with the National Institutes of Health's Guide for Care and Use of Laboratory Animals and the Animal Research: Reporting *in vivo* Experiments (ARRIVE) Guidelines. The protocol was approved by the Institutional Animal Care and Use Committee of the National University of Quilmes.

### Experimental Design

In the experiments conducted under LD conditions, animals were injected at ZT11 or ZT19 (ZT: zeitgeber time; ZT0: time of lights on; ZT12: time of lights off) with a dose of 20 mg/kg of LPS (*Escherichia coli* 0111:B4 serotype, Sigma-Aldrich, St. Louis, USA) or vehicle (VEH; saline solution). Mice were weighted 24 h before treatment, immediately before the injection and 24 h after treatment. For survival analyses mice were observed for 10 days after treatment, three times a day. Samples were collected 2 h after inoculation with LPS or VEH [except for the serum transfer experiment; see below]. Blood extraction was done under isofluorane anesthesia (5%; USP, Piramal Healthcare, India), using an equipment of gas anesthesia (SurgiVet®, USA). Tissue collection was done after euthanizing by rapid decapitation under isofluorane anesthesia, and all efforts were made to minimize suffering.

For experiments performed under circadian desynchronization, LPS or VEH was administered 3 weeks after the beginning of the CJL^6/2^ protocol (see below). Inoculation at ZT11 was done during the day (lights on) before the 6-h night, while ZT19 inoculation was done during the 12-h night before the mentioned day.

### Chronic Jet-Lag Protocol

The CJL schedule was previously designed by our group (Casiraghi et al., 2012) and consisted in a 6 h advance of the LD cycle every 2 days (CJL^6/2^); which was accomplished through a 6 h shortening of every second dark phase. Effective circadian desynchronization was evaluated by observation of a particular activity pattern which included two components of activity rhythms with periods of about 21 and 24.7 h. General activity was detected by infrared sensors connected to a computer interface that records activity counts every 5 min for posterior time-series analysis (Archron, Buenos Aires, Argentina).

### Body Temperature Analysis

For body temperature studies, individual photographs were taken using the *FlirOne*^®^ *Thermal Camera* (Flir Systems, Oregón, USA) coupled to a *Samsung S7 SmartPhone* (Samsung, Seoul, South Corea). This camera provides thermal images in a range of −20 to 120°C, with a 0.1°C resolution. Pictures were taken 1 h before, at the time of inoculation and then every 2 h, for 20 h. For taking the picture, the animal was taken out of the cage and the camera was fixed at the same height for all the experiments. Pictures were then analyzed with an algorithm programmed in the software *Matlab*, for automatic calculation of the maximum, minimum and average temperature, and localization of the maximum temperature inside the image. Curve analysis was done using the maximum temperature obtained for each time point, which was always observed in the head of the animal.

### Animal Perfusion and Brain Sections Obtaining

Mice were deeply anesthetized under gas isofluorane anesthesia (5%; USP, Piramal Healthcare, India) and perfused intracardially first with cold 0.01M phosphate buffer saline (PBS) and then with cold 4% paraformaldehyde in PBS. Brains were removed carefully, post-fixed overnight in 4% paraformaldehyde in PBS, cryoprotected in 10%, 20% and 30% sucrose in PBS for 24 h each solution; 30 μm thick coronal sections were cut with a freezing cryostat and collected in PBS.

### Immunohistochemistry for cFos in the Hypothalamus

Mice were perfused 2 h after LPS or VEH treatment as explained before. Free-floating brain coronal sections containing POA, SCN, and paraventricular nuclei (PVN) (15 sections/mouse) were blocked with 10% non-fat milk in PBS containing 0.4% Triton X-100 (PBS-T) and incubated with primary antisera raised in rabbit against cFos (Millipore, Massachusetts, USA; 1:1,000) diluted in PBS-T, for 24 h at 4°C. Sections were then treated using the avidin–biotin method with a *Vectastain Elite Universal kit* containing a biotin-conjugated secondary antibody, avidin and biotin-conjugated horseradish peroxidase (Vector Laboratories, Burlingame, CA) and Vector-VIP peroxidase substrate (SK-4600; Vector Laboratories, Burlingame, CA). Cell counting was performed with the *Fiji-ImageJ 1.51n* software (NIH, Maryland, USA) in hypothalamic sections, using the regions shown in Figures 2E–G.

### RNA Extraction and Real-Time PCR

Tissue from POA was carefully dissected 2 h after LPS injection under magnifying glass observation and collected in 100 μl of *TRIzol*^®^ reagent (Thermo Fisher Scientific, Massachusetts, USA). RNA was extracted according to the manufacturer's instructions. RNA solutions were quantified using a *NanoDrop1000* equipment (Thermo Fisher Scientific, Massachusetts, USA) and their integrity was evaluated by electrophoresis in a 1.2% agarose gel. cDNA was synthesized using 1,000 ng of total RNA, oligo(dT) primers and the *SuperScript*™ *III First-Strand Synthesis System* (Thermo Fisher Scientific, Massachusetts, USA). Gene amplification was performed on a *SmartCycler II Thermal Cycler Automated Real-Time PCR System* (Cepheid®, California, USA), using 25 μl of final reaction volume containing 1 μl of cDNA as template, 1X of *Master Mix qPCR* (Productos BioLógicos, Buenos Aires, Argentina) and the corresponding primers: TNFR1-F 5′-ACC AAG TGC CAC AAA GGA AC-3′, TNFR1-R 5′-ATT CTG GGA AGC CGT AAA GG-3′, TNF-F 5′-GAC AGT GAC CTG GAC TGT GG-3′, TNF-R 5′-GAG ACA GAG GCA ACC TGA CC-3′, HPRT-F 5′- TGT TGG ATA CAG GCC AGA C-3′, HPRT-R 5′ TGG CAA CAT CAA CAG GAC TC-3′. HPRT was used as the reference gene. The cDNA template was amplified in duplicate, with the following conditions: 95°C for 10 min, followed by 40 cycles of 95°C for 15 s and 60°C for 1 min. Then the melting curve was obtained between 60 and 95°C. Relative gene expression was analyzed using the 2^−ΔΔ*Ct*^ method.

### Flow Cytometry Analysis

Peritoneal exudate harvest was done 2 h after LPS injection. The peritoneal membrane was separated from the skin and 3 ml of cold *Dulbecco's Modified Eagle's Medium* (DMEM; Sigma-Aldrich, St. Louis, USA) was injected in the midline of the peritoneal cavity (separating the peritoneal membrane from the other tissues and organs). The fluid was gently agitated and then aspirated inserting the gauge in the left flank of the mouse. Spleen tissue was collected in DMEM (Sigma-Aldrich, St. Louis, USA). Following mechanical disruption, tissues were incubated with 1 mg/ml Colagenase IV (Thermo Fisher Scientific, Massachusetts, USA) in a shaker for 20 min at 37°C. Then, the collagenase activity was inhibited with 15% of fetal bovine serum (FBS; Internegocios, Buenos Aires, Argentina). The spleen cells were filtered with a 70 μm filter and red cells were lysed twice by incubation with ACK lysing buffer (NH_4_Cl 8,290 mg/L, KHCO_3_ 1,000 mg/L and EDTA 1 mM) in relation 1:9 for 7 min, and centrifuged at 400 g for 10 min at 4°C. The supernatant was discarded and 10^6^ cells were incubated for 40 min at room temperature with the antibodies for the corresponding surface antigen: F4/80 (catalog 123127), CD11b (catalog 557396), CD86 (catalog 105011), and CD206 (catalog 141705), or the corresponding isotype controls (PE rat IgG2a catalog 400507; PE IgG2b catalog 400607; PerCP7cy5.5 rat IgG2a catalog 4005312; APC rat IgG2a catalog 400511). All the antibodies were obtained from Biolegend, California, USA. Then, cells were washed with PBS-FBS 3%, centrifuged at 400 g for 7 min and maintained at 4°C protected from light until fluorescence detection by *BD FACSCalibur*^®^ *Flow Cytometer* (BD Biosciences, California, USA). Data was analyzed using the software *FlowJo 7.6* (BD Biosciences, California, USA).

### Serum Collection

Serum was collected 2 h after LPS injection. When the mouse lost its motor reflex due to the anesthesia, it was placed with its abdominal region upwards and blood was collected by cardiac puncture introducing a 25G gauge at 45° in the left ventricle. Then, blood was centrifuged for 10 min at 6,000 g, and serum was collected and immediately stored at −80°C.

### Serum Transfer Experiment

Mice were inoculated with 20 mg/kg of LPS at ZT11 or ZT19 or VEH at ZT11, and 2 h later (ZT13 or ZT21, respectively), blood was extracted and serum obtained, as explained before. The serum was filtered using a 0.45 μm syringe filter (Minisart®, Sartorius, Germany), and then injected intraperitoneally in naïve mice at ZT13. Each animal received 500 μl of serum, which resulted from serum extracted from two different mice.

### Cytokine Quantification Using Flow Cytometry

The serum concentration of the cytokines TNF-α, Interleuquin (IL)-6, IL-12p70, Interferon-γ (IFN-γ), IL-10 and the chemokine CCL2 were determined using the *Cytometric Bead Array Mouse Inflammation kit* (BD Biosciences, California, USA) according to the manufacturer's protocol. Samples were analyzed using a *BD FACSCalibur*^®^ *Flow Cytometer* (BD Biosciences, California, USA).

### Quantification of the Levels of Corticosterone in Serum

Serum samples coming from mice injected with VEH were diluted 1/10 and the ones coming from LPS injected mice were diluted 1/25 (so the assay was not saturated). Then, they were subjected to two successive extractions with dichloromethane, and analyzed by Radioimmunoassay (RIA).

### Quantification of the Levels of Endocannabinoids in Serum

The analysis was done using 500 μl of mice serum placed in a glass vial, to which 60 pmol of the 2-arachidonoyl glycerol (2-AG) standard (2-AGd5, Cayman Chemical, Michigan, USA) and 30 pmol of the Anandamide (AEA) standard (AEAd8, Cayman Chemical, Michigan, USA) were added. The standard mass was calculated doing a curve and obtaining the limits of detection (LOD) and quantification (LOQ). Two ml of ethyl acetate (HPLC quality) were added and mixed for 1 min using a vortex mixer. Then the sample was centrifuged at 1,400 g for 10 min. The organic phase was obtained and evaporated at room temperature under a stream of gas nitrogen. The samples were analyzed at the *Inmet Mass Spectrometry Service* (Ingeniería Metabólica SA, Santa Fé, Argentina), where they were resuspended in a 1:1 water:methanol solution, and analyzed using an LC-MS/MS equipment.

### Tissue Protein Extraction

Liver and spleen were dissected 2 h after LPS injection, and placed in 0.01 M PBS containing a protease inhibitor cocktail (P8340- Sigma Aldrich, St. Louis, USA). The tissue was homogenized and placed in ice for 15 min. Then, the sample was centrifuged for 15 min at 14,000 g, the supernatant was collected and stored at −80°C.

### Quantification of TNF-α Levels by ELISA

The levels of TNF-α in liver, spleen and serum (from mice subjected to the CJL^6/2^ protocol) were quantified using the *Mouse TNF (Mono/Mono) ELISA Set BD OptEIA* (BD Bioscience, California, USA). Liver proteins were diluted 1/20, spleen proteins 1/10 and serum samples 1/2 in dilution buffer prepared with FBS (Internegocios, Buenos Aires, Argentina). The assay was conducted according to the manufacturer's instructions, except for sample incubation which was done overnight. Plate absorbance was measured using *Cytation 5 Imaging Reader* (BioTek Instruments, Vermont, USA).

### Statistical Analysis

Data is presented as mean ± standard error of the mean (SEM). Differences between two groups were analyzed by unpaired Student's *t*-tests. The difference between more than two groups was analyzed by one or two-way analysis of variance (ANOVA) or the non-parametric Kruskal Wallis test. For temperature curves analysis two-way ANOVA repeated measures was used. *Post-hoc* pairwise comparisons were performed by means of a Bonferroni's test (after ANOVA) or Dunn's test (after Kruskal Wallis). Parametric tests were only used for data that fulfilled the assumptions of normality and homogeneity of the variances tests. *P*-values of 0.05 or less were considered to be statistically significant. GraphPad Prism7 and Infostat were used to perform these analyzes. El Temps software was used for cosinor analyzes. The complete statistical data is shown in the figure legends.

## Results

### Differential Thermoregulatory Response to High Doses of LPS

**Figure 1:**
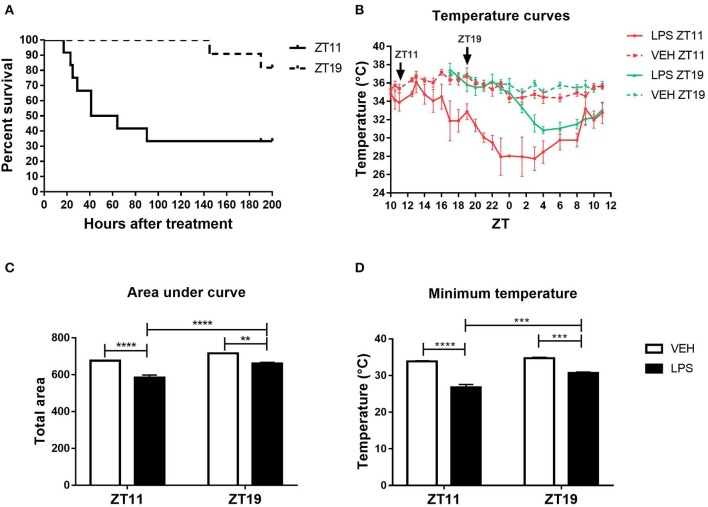
Daily differences in the thermoregulatory response to high doses of LPS. **(A)** Survival curves of animals injected intraperitoneally with 20 mg/kg of LPS or VEH at ZT11 or ZT19. **(B)** Temperature curves as a function of ZT of stimulated animals, **(C)** mean ± SEM of the area under each curve calculated since the time of inoculation to 20 h after treatment, and **(D)** mean ± SEM of the minimum temperature developed by each animal. **p < 0.01, ***p < 0.001, ****p < 0.0001. **(A)** Log-rank (Mantel Cox) test: *p* = 0.0085 (*n* = 12 per group). **(B)** Two-way ANOVA repeated measures (using the data since ZT19): *p* < 0.0001 for all the factors. **(C)** Two-way ANOVA: *p* < 0.0001 for time and treatment factors, *p* = 0.035 for interaction; followed by post-test: *p* < 0.001 LPS ZT11 vs. all groups, *p* < 0.002 LPS ZT19 vs. VEH ZT19, *p* = 0.022 VEH ZT11 vs. VEH ZT19. **(D)** Two-way ANOVA: *p* < 0.0001 for treatment factor, *p* = 0.0002 for time factor, *p* = 0.007 for interaction factor; followed by post-test: *p* < 0.0001 LPS ZT11 vs. VEH ZT11/19, *p* = 0.0004 LPS ZT11 vs. LPS ZT19, *p* = 0.0003 LPS ZT19 vs. VEH ZT19, *p* = 0.002 LPS ZT19 vs. VEH ZT11. B-D: *n* = 4 per group.

As we previously described, the mortality rate caused by high doses of LPS administered at the end of the day (ZT11) is higher than at the middle of the night (ZT19; Halberg et al., 1960; Marpegan et al., 2009). In order to further characterize the mechanisms responsible for this daily variation, we induced septic shock in mice by the intraperitoneal administration of 20 mg/kg of LPS. First of all, we confirmed that the inoculation at ZT11 induced a 77.66% of mortality while the administration at ZT19 led to 18.18% of mortality (Figure 1A; *p* = 0.0085). One characteristic of septic shock is the loss of appetite or anorexia (Granger et al., 2013). Indeed, we observed a higher weight loss in those animals injected at ZT11 (Supplementary Figure 1).

Another main feature of septic shock is the change in body temperature. Hypothermia is correlated with a poor prognosis in humans and in animal models (Fairchild et al., 2004; Peres Bota et al., 2004; Fonseca et al., 2016; Rumbus et al., 2017). Therefore, we analyzed the thermoregulatory response in animals injected with high doses of LPS at both times, using *FlirOne*^®^ thermal camera. The obtained temperature curves using the maximum values, which was always observed in the head of the animal, are shown in Figure 1B. Interestingly, those animals that received LPS at ZT11 developed a deeper hypothermia than those that were inoculated at ZT19 (*p* < 0.0001). As expected, the area under the temperature curve for animals stimulated at ZT11 was smaller than the ones inoculated at ZT19 (Figure 1C; *p* < 0.0001). Finally, we analyzed the minimum temperature attained by each animal and found that those injected at ZT11 showed a lower minimum temperature than those stimulated at ZT19 (Figure 1D; *p* < 0.0001). For both ZT11 and ZT19, inoculated animals reached the minimum temperature between 10 and 12 h after the stimulus. Supplementary Figure 2 shows representative thermal images obtained with *FlirOne*^®^ camera, for the groups injected with LPS (A) or VEH (B) at ZT11.

We can conclude that the daily difference in mortality rate is accompanied by a deeper hypothermia and higher weight loss, when septic shock is induced at the end of the day (ZT11).

### Central Nervous System Response to High Doses of LPS

The POA is one of the main brain regions involved in thermoregulation (Boulant, 1998; Morrison, 2016) even though other hypothalamic regions, as the PVN and SCN nuclei also participate in this response (Lu et al., 2001; Wanner et al., 2013; Guzmán-Ruiz et al., 2015). Moreover, we and others have found SCN and PVN activation in response to immune peripheral stimuli (Belevych et al., 2010; Paladino et al., 2014). In order to study if the peripheral signal triggered after LPS administration at ZT11 or ZT19 induced a differential hypothalamic activation, we analyzed the number of cFos positive cells (neuronal activation) in POA, SCN (core and shell regions) and PVN after septic shock induction.

**Figure 2:**
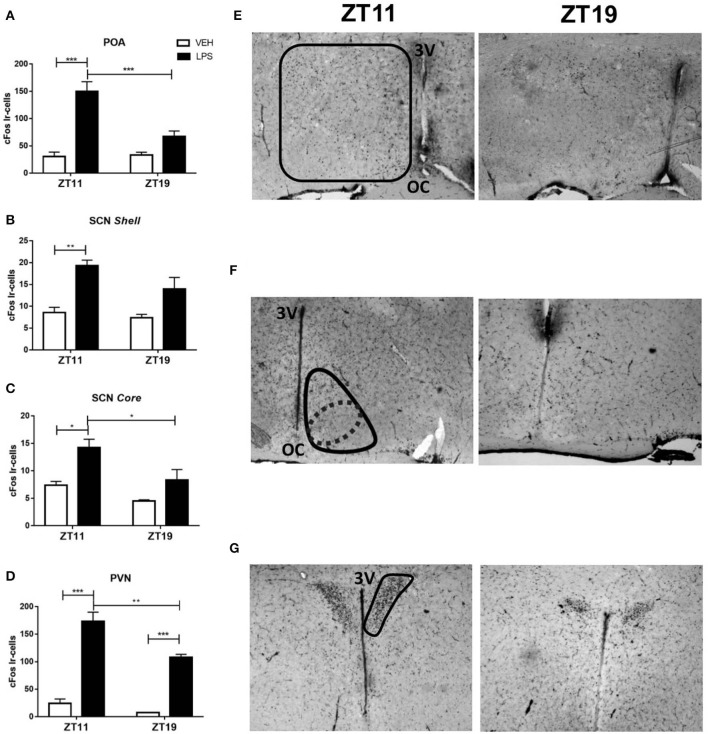
Central nervous system activation following LPS treatment. Mean ± SEM of the number of cFos immunoreactive (Ir) cells in **(A)** POA, **(B)** shell and **(C)** core of the SCN and **(D)** PVN of mice inoculated with 20 mg/kg of LPS or VEH at ZT11 or ZT19. Representative pictures of the immunohistochemistry showing **(E)** POA, **(F)** SCN, and **(G)** PVN regions. *p < 0.05, **p < 0.01, ***p < 0.001. **(A)** Two-way ANOVA: *p* < 0.0001 for treatment factor, *p* = 0.0116 for time factor, and *p* = 0.0077 for interaction; followed by post-test: *p* < 0.001 LPS ZT11 vs. VEH ZT11, *p* = 0.0003 LPS ZT11 vs. LPS ZT19, *p* = 0.0001 LPS ZT11 vs. VEH ZT19. **(B)** Two-way ANOVA: *p* = 0.0004 for treatment factor; followed by post-test: *p* = 0.0036 LPS ZT11 vs. VEH ZT11, *p* = 0.008 LPS ZT11 vs. VEH ZT19. **(C)** Two-way ANOVA: *p* = 0.018 for time factor and *p* = 0.005 for treatment factor; followed by post-test: *p* = 0.028 LPS ZT11 vs. VEH ZT11, *p* = 0.042 LPS ZT11 vs. LPS ZT19, *p* = 0.007 LPS ZT11 vs. VEH ZT19. **(D)** Two-way ANOVA: *p* < 0.0001 for treatment factor, and *p* = 0.005 for time factor; followed by post-test: *p* < 0.0001 LPS ZT11 vs. VEH ZT11, *p* = 0.002 LPS ZT11 vs. LPS ZT19, *p* = 0.0002 LPS ZT19 vs. VEH ZT19. *n* = 10 for LPS groups, *n* = 7 for VEH ZT11 and *n* = 4 for VEH ZT19. 3V: third ventricle. OC: optic chiasm. Solid lines delimit regions consider as POA **(E)**, SCN shell **(F)**, and PVN **(G)**. The dotted line delimits the region consider as SCN core **(F)**.

LPS administration at ZT11 induced an increase in the number of cFos inmunoreactive cells in POA (Figure 2A; *p* < 0.001) and in both core and shell regions of the SCN (Figures 2B,C; *p* = 0.0036 and *p* = 0.028). However, ZT19 inoculation did not significantly increase cFos expression in these brain regions. Additionally, in PVN, while LPS activated neurons at both times, cFos expression was higher at ZT11 compared to ZT19 (Figure 2D; *p* = 0.002). Representative images of the immunohistochemistries are shown in Figures 2E–G.

**Figure 3:**
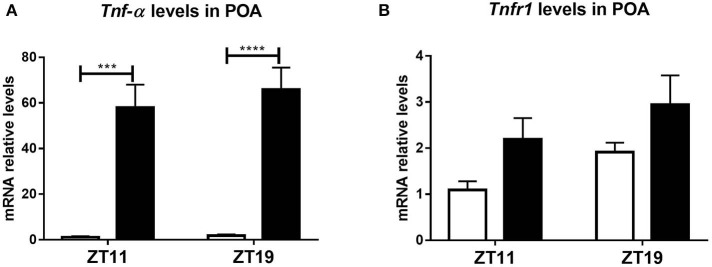
*Tnf-*α and *Tnfr1* mRNA levels in POA. Mean ± SEM of **(A)** *Tnf-*α and **(B)** *Tnfr1* mRNA expression in POA, in mice injected with 20 mg/kg of LPS or VEH at ZT11 or ZT19. ***p < 0.001, ****p < 0.0001. **(A)** Two-way ANOVA: *p* < 0.0001 for treatment factor; followed by post-test: *p* = 0.0001 LPS ZT11 vs. VEH ZT11, *p* < 0.0001 LPS ZT19 vs. VEH ZT19. **(B)** Two-way ANOVA: *p* = 0.014 for treatment factor. *n* = 6 for all groups, except LPS ZT19 *n* = 5.

This central activation evidenced a differential hypothalamic response to peripheral LPS administration. TNF-α and its type 1 receptor TNFR1 are expressed in the brain (Botchkina et al., 1997; Sadki et al., 2007; Camara et al., 2015) and its expression increase after LPS injection (Gatti and Bartfai, 1993; Layé et al., 1994). Furthermore, there are many evidences of the role of these molecules, both in the immune-circadian communication (Cavadini et al., 2007; Duhart et al., 2013; Paladino et al., 2014) and in the hypothermic response to high doses of LPS (Leon et al., 1998; Nautiyal et al., 2009). In order to study if the TNF-α pathway is related to the daily variation observed in the hypothermic response and the hypothalamic neuronal activation, we hypothesized that the peripheral inflammatory signal induces a differential expression of this cytokine or its receptor in the POA according to the time of LPS inoculation. Indeed, we found that both the expression of TNF-α and TNFR1 was induced in the POA, but this induction was independent on the time when LPS was administered (Figures 3A,B; A: *p* < 0.0001; B: *p* < 0.0143).

In conclusion, these results show that the signal elicited by peripheral administration of LPS at ZT11 triggers activation of hypothalamic regions in a time-dependent manner. However, *Tnf-*α and *Tnfr1* expression in POA was induced by LPS independently of the time of administration.

### Inflammatory Response in Peritoneum and Peripheral Tissues

Macrophages are an essential component of the immune response to infections, including sepsis (Dahdah et al., 2014; Cheng et al., 2018). In order to analyze if this immune response is related to the differences described above, we studied the percentage and the activation levels of peritoneal macrophages, which may be directly affected by the inoculation, and macrophages present in the spleen, that is one of the secondary immune organs which acts as the main filter of pathogens and antigens present in blood (Bronte and Pittet, 2013).

**Figure 4:**
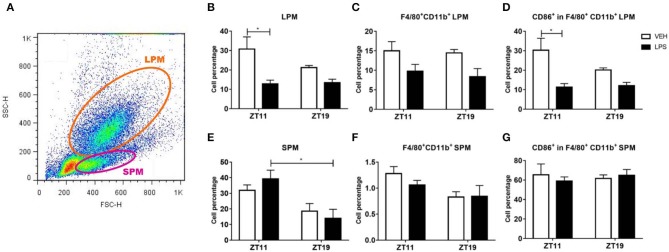
Macrophage percentage and activation level in peritoneal exudate. **(A)** Dot plot showing size (FSC) vs. membrane complexity (SSC) of peritoneal cells from mice injected with 20 mg/kg of LPS or VEH at ZT11 or ZT19. Mean ± SEM of **(B,E)** total cell percentage, **(C,F)** F4/80^+^-CD11b^+^ cell percentage and **(D,G)** CD86^+^-F4/80^+^-CD11b^+^ cell percentage, in the LPM (**B–D**) and the SPM (**E–G**) subset. **(A)** The orange line delimits cells consider as LPMs and the violet line delimits cells consider as SPMs. *p < 0.05. **(B)** Kruskal Wallis test *p* = 0.019, followed by post-test: *p* < 0.05 LPS ZT11 vs. VEH ZT11. Two-way ANOVA: **(C)** *p* = 0.015 for treatment factor; **(D)** *p* = 0.004 for treatment factor, followed by post-test: *p* = 0.012 LPS ZT11 vs. VEH ZT11; **(E)** *p* = 0.004 for time factor, followed by post-test: *p* = 0.035 LPS ZT11 vs. LPS ZT19; **(F)** *p* = 0.028 for time factor. *n* = 5 for LPS ZT11, *n* = 4 for VEH ZT11 and *n* = 3 for ZT19 groups.

As expected, we observed two different peritoneal macrophages subsets: small peritoneal macrophages (SPMs) and large peritoneal macrophages (LPMs). These subsets differ in their size and in the complexity of their membranes, among other features, as it is shown in the dot plot of Figure 4A.

We found that the cell percentage of LPMs decreased after inoculation of LPS at ZT11, but not at ZT19 (Figure 4B; *p* = 0.019). Additionally, we specifically analyzed the percentage of macrophages in the LPM subset, identified by the surface markers F4/80 and CD11b, and found a reduction of this subset after inoculation with LPS at both times (Figure 4C; *p* = 0.0151). We also studied activation of the macrophage subset analyzing the expression of the surface molecule CD86, and observed that it was decreased only after ZT11 stimulation (Figure 4D; *p* = 0.0119).

When we analyzed the SPM subset, we found that there was no alteration in cell percentage due to the treatment, but there was a daily variation of the basal (vehicle) percentages, showing higher levels at ZT11 compared to ZT19 (Figure 4E; *p* = 0.0346). A similar result was observed when we studied the percentage of macrophages (F4/80^+^-CD11b^+^) in the SPM subset (Figure 4F; *p* = 0.0280). However, we did not observe time-dependent differences in the activation (CD86^+^) of this subset of macrophages (Figure 4G).

**Figure 5:**
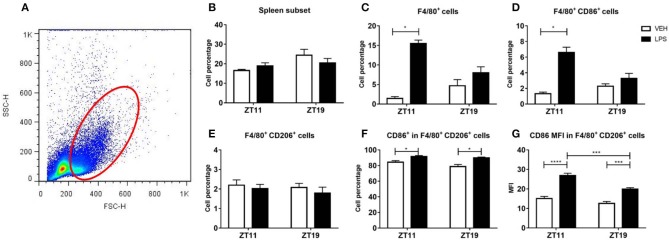
Macrophage percentage and activation level in spleen. **(A)** Dot plot showing size (FSC) vs. membrane complexity (SSC) of spleen cells isolated from mice injected with 20 mg/kg of LPS or VEH at ZT11 or ZT19. Mean ± SEM of **(B)** total cell percentage, **(C)** F4/80^+^ cell percentage, **(D)** F4/80^+^-CD86^+^ cell percentage, **(E)** F4/80^+^-CD206^+^ cell percentage, **(F)** CD86^+^-F4/80^+^-CD206^+^ cell percentage, and **(G)** mean fluorescence intensity of CD86 in F4/80^+^-D206^+^ cells, in the spleen subset studied. **(A)** The red line delimits the cell subset studied. *p < 0.05, ***p < 0.001, ****p < 0.0001. Kruskal Wallis test: **(C)** *p* = 0.0003, followed by post-test: *p* < 0.05 LPS ZT11 vs. VEH ZT11; **(D)** *p* = 0.001, followed by post-test: *p* < 0.05 LPS ZT11 vs. VEH ZT11; **(F)** *p* = 0.003, followed by post-test: *p* < 0.05 LPS ZT11 vs. VEH ZT11 and LPS ZT19 vs. VEH ZT19. **(G)** Two-way ANOVA: *p* < 0.0001 for treatment factor, *p* = 0.0002 for time factor and *p* = 0.040 for interaction, followed by post-test: *p* < 0.0001 LPS ZT11 vs. VEH ZT11, *p* = 0.0007 LPS ZT11 vs. LPS ZT19, and *p* = 0.0004 LPS ZT19 vs. VEH ZT19; *n* = 6 per group.

Regarding the spleen (Figure 5A), the cell percentage in the analyzed subset was similar in response to treatment and time (Figure 5B). We observed that in this cell subset, treatment with LPS at ZT11, but not at ZT19, induced an increase in total macrophage percentage, identified as F4/80^+^ cells (Figure 5C; *p* < 0.05), as well as in the activated (F4/80^+^CD86^+^) macrophage percentage (Figure 5D; *p* < 0.05). Nevertheless we did not find differences in CD86 expression, calculated as the mean fluorescence intensity (MFI; data not shown).

We also studied the percentage and level of activation of type M2 macrophages (associated with anti-inflammatory and homeostatic functions), which are identified by the surface marker CD206. Despite we did not find differences in cell percentage of type M2 macrophages (F4/80^+^-CD206^+;^ Figure 5E), we observed a significant activation (F4/80^+^ CD206^+^ CD86^+^) after stimulation with LPS at both times, evidenced by the percentage of cells (Figure 5F; *p* = 0.0028), and by an increase of CD86 MFI (Figure 5G; *p* < 0.0001). Interestingly, the MFI of CD86 was higher after inoculation at ZT11 than at ZT19 (*p* = 0.0007).

Moreover, we analyzed TNF-α levels in spleen and liver tissue. As mentioned before, the spleen has an important role in innate immunity, while the liver participates in many innate immune functions, as the acute phase response, in which TNF-α has an important role (Parker and Picut, 2005; Kubes, 2016). TNF-α protein levels in both liver and spleen were not modified by the treatment nor the time of administration (Supplementary Figures 3A,B).

In conclusion, once again we observed differences between the response elicited by LPS at ZT11 or ZT19. In the peritoneum we found that both total cell percentage and macrophage activation in the LPM subset decreased after LPS stimulation at ZT11. In spleen, we observed an increase in total and activated macrophage percentage in response to LPS administration at ZT11, and increased activation levels of M2 macrophages.

### Differences in Serum Composition of Animals Stimulated at ZT11 or ZT19

**Figure 6:**
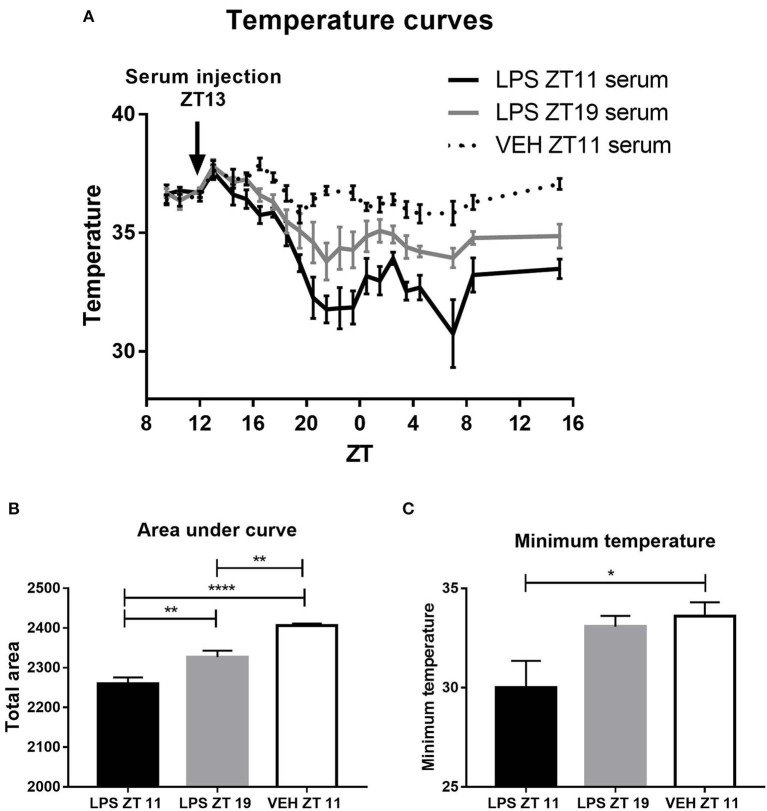
Thermoregulatory response to inoculation with serum from LPS injected animals. Animals were inoculated at ZT13 with serum from animals stimulated with LPS at ZT11 or ZT19, or VEH at ZT11. **(A)** Temperature curves as a function of ZT, mean ± SEM of **(B)** the area under each curve calculated since the time of inoculation to 20 h after treatment, and **(C)** the minimum temperature developed by each animal. *p < 0.05, **p < 0.01, ****p < 0.0001. **(A)** Two-way ANOVA repeated measures: *p* < 0.0001 for time factor and interaction and *p* = 0.0001 for treatment factor. One-way ANOVA: **(B)** *p* < 0.0001, followed by post-test: *p* < 0.001 LPS ZT11 vs. VEH ZT11, *p* = 0.0023 VEH ZT11 vs. LPS ZT19 and *p* = 0.0086 LPS ZT11 vs. LPS ZT19; **(C)** *p* = 0.0325, followed by post-test: *p* = 0.0467 LPS ZT11 vs. VEH ZT11; *n* = 6 per group.

As we found daily differences both in the immune and the thermic response, we decided to evaluate changes in serum composition in septic animals which could be related to this time-dependent response. With this purpose animals were inoculated with 20 mg/kg of LPS at ZT11 or ZT19, and 2 h later (ZT13 or ZT21, respectively), blood was collected to obtain the serum. Then, these sera were injected to naïve animals at ZT13 and body temperature was monitored. As shown in the temperature curves and the area under the curve, animals which received the serum coming from animals stimulated at ZT11 developed a deeper hypothermia than those that received the serum of animals injected at ZT19 (Figure 6A: *p* = 0.0001; Figure 6B: *p* < 0.0001). Additionally, mice injected with the serum of those inoculated at ZT11 developed a lower minimum temperature (Figure 6C, *p* = 0.0325). These results show that the differential hypothermic response is due to some molecule or molecules present in the serum of septic animals.

**Figure 7:**
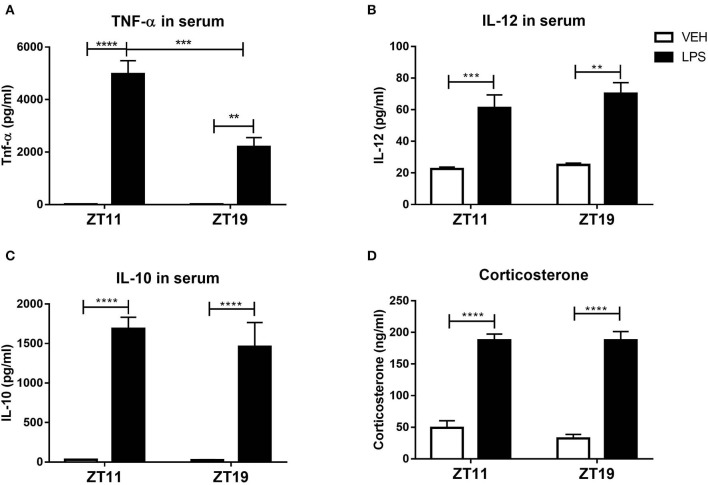
Serum composition of septic mice. Mean ± SEM of **(A)** TNF-α, **(B)** IL-12, **(C)** IL-10, and **(D)** corticosterone levels in the serum of mice injected with 20 mg/kg of LPS or VEH at ZT11 or ZT19. **p < 0.01, ***p < 0.001, ****p < 0.0001. Two-way ANOVA: **(A)** *p* < 0.0001 for treatment factor, *p* = 0.0011 for time factor and *p* = 0.0012 for interaction, followed by post-test: *p* < 0.0001 LPS ZT11 vs. VEH ZT11, *p* = 0.0005 LPS ZT11 vs. LPS ZT19, *p* = 0.0059 LPS ZT19 vs. VEH ZT19; **(B)** *p* < 0.0001 for treatment factor, followed by post-test: *p* = 0.0008 LPS ZT11 vs. VEH ZT11 and *p* = 0.0011 LPS ZT19 vs. VEH ZT19; **(C)** *p* < 0.0001 for treatment factor, followed by post-test: *p* < 0.0001 LPS vs. VEH for both times. **(A–C)**: *n* = 5 for ZT11 groups, *n* = 3 for LPS ZT19 and *n* = 4 for VEH ZT19. **(D)** Two-way ANOVA: *p* < 0.0001 for treatment factor, followed by post-test: *p* < 0.0001 for LPS ZT11/19 vs. VEH ZT11/ZT19; *n* = 7 per group.

Therefore, we analyzed serum composition of mice inoculated at both times. One the main characteristics of septic shock is the development of an exacerbated inflammatory response characterized, among other things, by an increase of cytokines levels (Cinel and Opal, 2009). Indeed, the levels of the pro-inflammatory cytokines TNF-α and IL-12, and of the anti-inflammatory cytokine IL-10, were increased 2 h after treatment with LPS at both times (Figures 7A–C; *p* < 0.0001). It is striking that only the levels of TNF-α were higher in those mice treated at ZT11 compared to the ones inoculated at ZT19 (*p* = 0.0005). IFN-γ was not detectable in none of the groups.

In addition, the levels of glucocorticoids, such as corticosterone, which have immunosuppressant functions (Stahn and Buttgereit, 2008), are increased in sepsis (Kwon et al., 2007; Yamashita et al., 2017). We found that the levels of corticosterone in serum were increased after LPS treatment, independently of inoculation time (Figure 7D, *p* < 0.0001).

An alteration of endocannabinoid levels in response to inflammatory processes, including sepsis, has been reported (Szafran et al., 2015; Turcotte et al., 2015). Furthermore, they were also related to the hypothermia induced by high doses of LPS (Schindler et al., 2017). However, serum levels of the endocannabinoid 2-AG (which was reported to be altered in sepsis models) were not modified after stimulation at both times (Supplementary Figure 4). Moreover, the endocannabinoid AEA was not detectable in none of the conditions.

All together, these results show that the composition of the serum of animals injected with LPS at the end of the day (ZT11) is different from the one of mice stimulated in the middle of the night (ZT19), and elicits a different response in naïve animals. Surprisingly, when we studied serum composition we found that the only molecule that increased differentially was TNF-α, making it a strong candidate for the time-dependent modulation of septic responses.

### Response to High Doses of LPS in TNFR1 KO Animals

As we found a difference in the levels of TNF-α in serum of animals stimulated with LPS at ZT11 compared to the ones inoculated at ZT19, we studied the response to high doses of LPS in animals deficient for the TNF-α receptor type 1 (TNFR1 KO).

**Figure 8:**
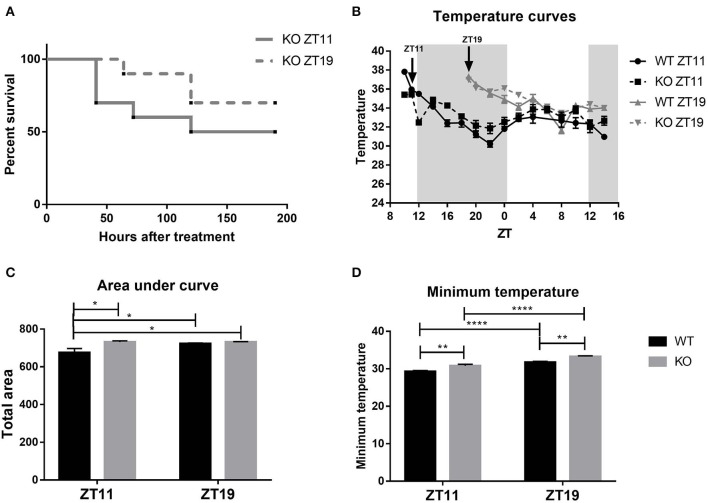
TNFR1 KO mice daily response to high doses of LPS. **(A)** Survival curves of TNFR1 KO animals injected intraperitoneally with 20 mg/kg of LPS at ZT11 or ZT19. **(B)** Temperature curves as a function of ZT of stimulated animals, mean ± SEM **(C)** of the area under each curve calculated since the time of inoculation to 20 h after treatment, and **(D)** of the minimum temperature developed by each animal. *p < 0.05, **p < 0.01, ****p < 0.0001. **(A)** Log-rank (Mantel Cox) test: non-significant. **(B)** Two-way ANOVA repeated measures: *p* < 0.0001 for all the factors. Two-way ANOVA: **(C)** *p* = 0.0163 for strain factor, followed by post-test: *p* = 0.0163 WT ZT11 vs. KO ZT11, *p* = 0.0447 for WT ZT11 vs. WT ZT19, *p* = 0.0176 for WT ZT11 vs. KO ZT19; **(D)** *p* < 0.0001 for time and strain factor, followed by post-test: *p* < 0.0001 WT ZT11 vs. WT/KO ZT19 and KO ZT11 vs. KO ZT19, *p* = 0.0064 WT ZT11 vs. KO ZT11, *p* = 0.0058 WT ZT19 vs. KO ZT19. **(A)** *n* = 10 for both groups. **(B–D)**: *n* = 12 for WT ZT11, *n* = 11 for WT ZT19, *n* = 10 for both KO groups.

When we compared the survival curves obtained after the inoculation with LPS of TNFR1 KO animals at ZT11 or ZT19, we found that TNFR1 deficiency significantly decreased the daily difference in the mortality rate, with a ≈50% of mortality rate for the ZT11 group and ≈30% for the ZT19 group (Figure 8A, *p* = ns).

Before analyzing the thermic response to high doses of LPS, we studied the temperature rhythms of these animals, because we did not have any data available. WT mice exhibit a body temperature rhythm with higher temperatures during the night (Supplementary Figures 5A,B; ANOVA *p* < 0.0001; Cosinor *p* = 0.003). However, body temperature values in TNFR1 KO mice did not adjust to a 24 h cosine waveform (Supplementary Figure 5D), although there were significant differences among time points (Supplementary Figure 5C; ANOVA *p* = 0.0078).

When we analyzed the thermic response to high doses of LPS, we found that TNFR1 KO mice developed hypothermia, but once again the time-of-day dependency observed in WT was lost in KO animals for both temperature curves (Figure 8B) and area under the curve (Figure 8C; *p* = 0.0163). Finally, Figure 8D shows that TNFR1 KO mice inoculated at ZT11 did develop a lower minimum temperature than those stimulated at ZT19, although WT animals reached even lower temperatures compared to the corresponding KO group (*p* < 0.0001), with lower values at ZT11 (p <0.01). Moreover, cFos levels in POA, SCN core and shell, and PVN regions of TNFR1 KO mice injected at both times were lower than those from WT animals inoculated at ZT11 (Supplementary Figure 6; *p* < 0.05). Moreover, we did not observe differences between the levels of those TNFR1 KO mice injected at ZT11 and ZT19.

These results show once again that the TNF-α signaling pathway is involved in the differential response to high doses of LPS. However, TNFR1 KO mice still present some daily differences in response to endotoxin, evidencing that TNF-α signaling through TNFR1 is not the only modulatory pathway responsible for the daily variation in the response to sepsis.

### Effects of Circadian Desynchronization on the Response to High Doses of LPS

Previous studies have shown that circadian desynchronization conditions lead to an increased susceptibility to LPS (Castanon-Cervantes et al., 2010; Fonken et al., 2013; Guerrero-Vargas et al., 2014). This highlights the influence of the circadian system in the response to high doses of LPS. To deepen this idea, we analyzed the daily response to high doses of LPS in animals maintained under a protocol of experimental chronic jet-lag (CJL^6/2^).

**Figure 9:**
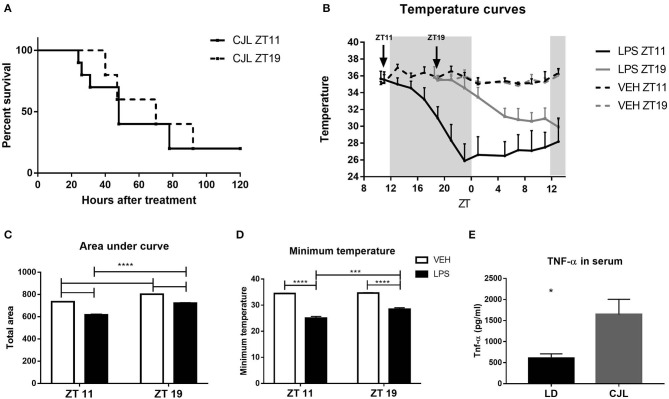
Effects of circadian desynchronization on the response to high doses of LPS. **(A)** Survival curves of animals subjected to CJL injected intraperitoneally with 20 mg/kg of LPS at ZT11 or ZT19. **(B)** Temperature curves as a function of ZT of stimulated animals. Mean ± SEM of **(C)** the area under each curve calculated since the time of inoculation to 20 h after treatment, **(D)** the minimum temperature developed by each animal, **(E)** TNF-α levels in the serum of animals subjected to CJL or LD conditions, injected with 20 mg/kg of LPS at ZT11. *p < 0.05, ***p < 0.001, ****p < 0.0001. **(A)** Log-rank (Mantel Cox) test: non-significant; *n* = 10 for both groups. **(B)** Two-way ANOVA repeated measures: *p* < 0.0001 for all the factors. Two-way ANOVA: **(C)** *p* < 0.0001 for time and treatment factors, *p* = 0.0099 for interaction, followed by post-test: *p* < 0.0001 LPS ZT11 vs. all groups and LPS ZT19/VEH ZT11 vs. VEH ZT19; **(D)** *p* < 0.0001 for time factor, *p* = 0.0049 for treatment factor, *p* = 0.0109 for interaction, followed by post-test: *p* < 0.0001 LPS vs. VEH for both times, *p* = 0.0002 LPS ZT11 vs. LPS ZT19. **(B–D)** *n* = 10 for LPS groups and *n* = 5 for VEH groups. **(E)** Unpaired T-test: *p* = 0.0142; *n* = 5 for CJL and *n* = 6 for LD.

Interestingly, desynchronized animals exhibited an 80% of mortality rate, independently of time of inoculation (ZT11 or ZT19), showing very similar survival curves (Figure 9A). This mortality rate is similar to the one observed in mice maintained under LD conditions injected at ZT11. We also studied weight loss after LPS treatment of desynchronized animals and found no difference between both groups of mice maintained under CJL (Supplementary Figure 7).

Mice kept under CJL conditions showed an arrhythmic pattern in the daily variation of body temperature (Supplementary Figure 5E). Additionally, as expected, the cosinor adjustment was not significant (Supplementary Figure 5F). When we studied the hypothermic response to immune stimulation, we observed that desynchronized animals which received LPS at ZT11 developed again a deeper response than those injected at ZT19 (Figure 9B, *p* < 0.0001). Furthermore, the area under the curve and minimum temperature were lower in mice inoculated at ZT11 compared to those inoculated at ZT19 (Figures 9C,D; C: *p* = 0.0099; D: *p* = 0.0109). Interestingly, when we compared these values with the ones observed in animals maintained in LD conditions, we found that animals in CJL developed lower minimum temperatures (LD ZT11: 28.06 ± 0.73, *n* = 10; LD ZT19: 31.36 ± 0.5, *n* = 10; CJL ZT11: 25.08 ± 0.6; CJL ZT19: 28.48 ± 0.53; two-way ANOVA: *p* < 0.0001 for light condition and *p* < 0.0001 for time; data not shown).

We also measured TNF-α levels in the serum of desynchronized animals injected at ZT11, and we found that those mice had increased levels of this cytokine compared to animals inoculated at ZT11 in LD conditions (Figure 9E; *p* = 0.014).

In conclusion, circadian desynchronization worsens the response to high doses of LPS and the differential response in the mortality rate is lost under this condition. Furthermore, we observed again that TNF-α is related to the circadian response in sepsis.

## Discussion

**Figure 10:**
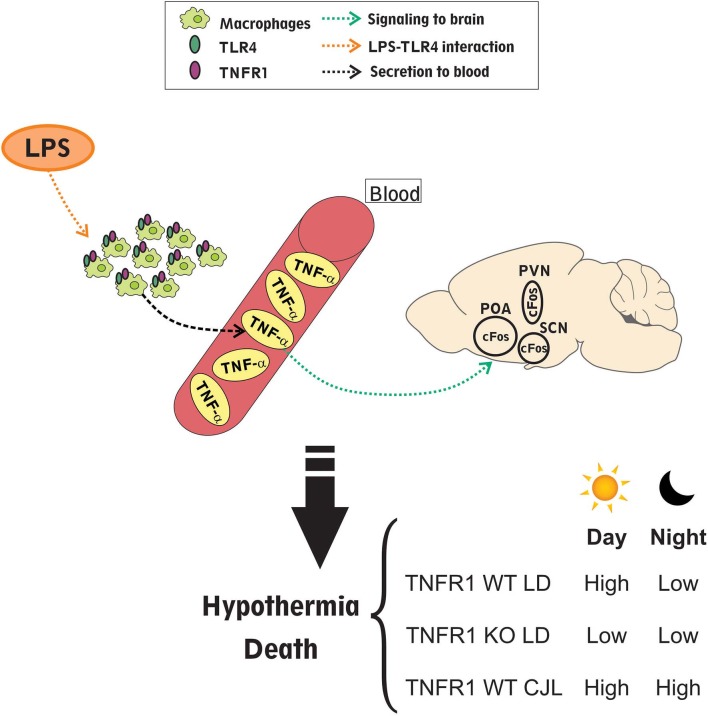
Daily differences in the response to LPS induced septic-shock. Intraperitoneal LPS administration acts through TLR4 on macrophages which can secrete cytokines to circulation, especially TNF-α; with higher levels of it after injection at ZT11 compared to ZT19 and also in animals subjected to CJL. Additionally, either LPS or cytokines can signal to the central nervous system through LPS or cytokine receptors present in the brain-blood barrier or through receptors present in the vagus nerve. This signaling can be responsible for hypothalamic activation (studied as cFos inmunoreactive cells), which again is higher at ZT11. Finally, this activation may induce the hypothermic response that is differential at ZT11 compared to ZT19, and again deeper in those mice kept in CJL conditions. Interestingly, TNFR1 KO mice did not show daily differences in the mortality rate, hypothermia levels and hypothalamic activation, which demonstrates that TNF-α signaling pathway is related to the septic daily response.

In the present study we have shown the importance of the circadian clock and its synchronization in the strength of the septic response. Figure 10 summarizes the main findings of this work. We found that LPS administered intraperitoneally may act on macrophages inducing the secretion of inflammatory cytokines, such as TNF-α, which pass to blood. Interestingly, the levels of TNF-α in serum are higher in those mice inoculated at ZT11 compared to ZT19, and in those subjected to CJL. TNF-α can signal to the central nervous system through receptors present in the brain-blood barrier (Mallard, 2012; Johnson et al., 2018) or by nervous pathways, as the vagus nerve (Bonaz et al., 2017), activating hypothalamic structures, as the POA, SCN and PVN, and the HPA axis (glucocorticoid secretion). Hypothalamic activation (cFos expression), again, was exacerbated at ZT11 and we hypothesized that it may be related to the development of hypothermia which was also deeper after administration of LPS at this time. Therefore, the differential mortality rate observed after inoculation of high doses of LPS at ZT11 or ZT19 is accompanied by a differential hypothermic response and induction of TNF-α levels. These results are consistent with data from other groups that showed that high doses of LPS induced hypothermia, which corresponds to more severe forms of inflammation (Nautiyal et al., 2009; Stewart et al., 2010; Garami et al., 2018). In line with our results, Silver et al. observed a deeper hypothermia in mice subjected to cecal ligation and puncture at ZT19 in comparison to the ones operated at ZT7, which correlated with an earlier mortality (Silver et al., 2012). The difference in the time of greater severity compared to the high doses of LPS model can be due to a difference in the kinetics and severity with which the septic shock is triggered in both models.

Therefore, the severity of septic shock may be due to the pro-inflammatory response elicited by LPS, which is related to TNF-α signaling through TNFR1, as confirmed by this work. These results are in line with the ones reported by Hrushesky et al., in which they showed that peripheral administration of TNF-α at different times induced a daily variation in the mortality rate similar to that observed with LPS administration (Hrushesky et al., 1994). Here we show, for the first time, that the lack of TNFR1 not only increases mice survival after septic shock induction (which was previously observed by Pfeffer et al., 1993; Leon et al., 1998; Guo et al., 2009), but also abolishes the daily difference in mortality, hypothermia and neuronal activation in response to high doses of LPS, as can be observed in Figure 10. It was previously reported that TNF-α is related to the pyrogenic response to inflammation (Stefferl et al., 1996; Luheshi et al., 1997). However, previous studies suggested that it is also a cryogenic cytokine, capable of reducing body temperature, since its systemic administration decreases the fever induced by LPS (Long et al., 1992; Klir et al., 1995). Even though in this study TNFR1 KO mice did not show differences in the temperature curves, the ones injected at ZT11 developed lower minimum temperatures than those injected at ZT19.

Hypothalamic neuronal circuits allow to keep body temperature homeostasis in the presence of different stimuli as environmental temperature changes or even immune challenges (Morrison, 2016). One of the main thermosensitive regions is the POA, which is able to sense its own temperature as well as the one from peripheral tissues that have thermoreceptors as the skin, different organs and the spinal cord (Boulant, 1998; Zhao et al., 2017; Tan and Knight, 2018). Other groups have reported neuronal activation of the POA in mice which developed fever or hypothermia (Yoshida et al., 2005; Uchida et al., 2014). Furthermore, other hypothalamic regions, as the SCN and PVN, are capable of receiving and modulating thermal signals (Lu et al., 2001; Wanner et al., 2013; Guzmán-Ruiz et al., 2015). Immune peripheral signals can reach the central nervous system by humoral or nervous pathways, which in turn can induce glial activation and the secretion of pro- or anti-inflammatory molecules (Meneses et al., 2019). Previous studies have shown neuronal activation in response to LPS (Hare et al., 1995; Marpegán et al., 2005; Paladino et al., 2010, 2014), but this work provides the first indication of a circadian modulation in hypothalamic responses to high doses of this molecule. As shown in this work, the greater activation of the hypothalamic regions studied correlates with the higher mortality rate and hypothermic response, and is related to TNF-α signaling pathway since TNFR1 KO mice showed lower cFos expression and daily difference was abolished. These results reinforce the idea that this particular molecule is central to the differential septic response. Nevertheless, we observed that both *Tnf-*α and *Tnfr1* expression are induced in POA in response to septic shock, independently of the time of LPS inoculation, which suggests that these molecules are related to the thermoregulatory mechanism but not to the daily difference observed in this hypothalamic region. Until now, there were no reports about the expression of this cytokine and its receptor in this hypothalamic region in response to high doses of LPS. This suggests that TNF-α signaling may not be the only pathway responsible for septic shock. In patients, treatment with TNF-α antagonists induced varied and controversial results that may be due to the complexity and diversity in the response to septic shock (Fisher et al., 1993; Lv et al., 2014). This complexity in the clinical response can be related to a TNF-α differential induction according to the time when septic shock is triggered, as observed in our studies.

Moreover, circadian synchronization seems to be an important factor for the response to septic shock. Here we show that the exacerbation in the mortality rate and hypothermic response is accompanied by an increase in the serum levels of TNF-α in desynchronized mice stimulated at ZT11 compared to those kept under LD conditions. Additionally, previous studies from our group have shown that in constant dark conditions, the daily difference in the response to high doses of LPS is lost and the survival percentages are similar to those observed after ZT11 inoculation in LD conditions (Marpegan et al., 2009). Other groups have shown that mice deficient for the circadian genes Per2 or Clock, are more resistant to septic shock, the daily difference in the mortality rate is lost and correlates with lower levels of inflammatory cytokines in serum (Liu et al., 2006b; Wang et al., 2016); which highlights again the importance of a functional circadian system. In the present work, we found that desynchronized mice had a high mortality rate (80%) independently from the time of inoculation. Carlson and Chiu also observed a lower survival in animals subjected to cecal ligation and puncture kept under constant light conditions (disruptive conditions of the circadian cycle) after the surgical intervention (Carlson and Chiu, 2008). Moreover, it has been shown that mice subjected to four acute changes (one per week) of the LD cycle have a higher mortality rate (Castanon-Cervantes et al., 2010). This suggests that the absence of temporal external cues as well as desynchronization can reduce the survival percentage to sepsis.

Furthermore, in the present work we still found a daily difference in the hypothermic response to high doses of LPS in desynchronized mice. This result suggests that the mechanism responsible for the development of the hypothermic response keeps its synchrony in mice subjected to this illumination scheme. This can occur because, in this CJL schedule, some of the components of the circadian system may undergo relative coordination and become partially synchronized to an external cue which is not strong enough to establish a steady synchronization (Casiraghi et al., 2012). However, as has been shown in this work and in previous studies (Castanon-Cervantes et al., 2010), the minimum temperatures developed by the animals kept under CJL conditions were lower than those observed in animals kept under a LD cycle.

To further analyze the inflammatory response in this model, we studied macrophage percentage and level of activation in the peritoneum and spleen. As mentioned before, peritoneal macrophages can be classified according to their morphology in LPMs and SPMs (Cassado Ados et al., 2015). The LPMs constitute approximately 90% of the peritoneal macrophages in non-stimulated animals, but their levels decrease quickly in response to stimuli like LPS or tioglicolate administered intraperitoneally. Moreover, these cells express high levels of the LPS receptor TLR-4 (Toll like-receptor 4), and co-stimulatory molecules (as CD86). It has been reported that due to the excessive inflammation produced during sepsis, a great number of factors that promote macrophage apoptosis are induced (Zhu et al., 2009; Luan et al., 2015). Additionally, in mice experimentally infected with *Enterococcus faecium*, it has been shown that peritoneal macrophages are important for the containment of the infection and that its depletion delays the clearance of the pathogen (Leendertse et al., 2009). Moreover, the decrease in the levels of LPMs may be related to their migration to other tissues (Okabe and Medzhitov, 2014). In line with these reports, we found that the levels of LPMs decrease in response to high doses of LPS. In particular, the total cell and activated macrophage percentage decreased more after LPS administration at ZT11 than at ZT19. There are evidences that the decrease in the levels of LPMs in response to an inflammatory stimulus is accompanied by the arrival of inflammatory monocytes to the peritoneal cavity (Cassado Ados et al., 2015). Moreover, it has been shown that the levels of SPMs increase after 2 days in response to low doses of LPS stimulation and that the monocytes that arrive to the cavity differentiate into this subset of macrophages (Ghosn et al., 2010). Additionally, *in vitro* studies have shown that SPMs develop a pro-inflammatory profile in response to LPS, since they produce high levels of pro-inflammatory cytokines, among them TNF-α (Cain et al., 2013). In this work, we observed a daily variation in the percentage of this subset of macrophages, with higher levels at ZT11; which can be related to the higher levels of TNF-α found in serum in response to LPS inoculation at ZT11.

Spleen macrophages are also an important component of the innate immune response (Liu et al., 2006a). We found an increase in the percentage and activation of these cells after LPS administration at ZT11, which was not observed after inoculation at ZT19. Other groups have shown an increase in the expression of CD86 in these macrophages in response to LPS (Liu et al., 2006a), but, until now, there was no data on time-dependent responses. Type M1 macrophages are characterized by pro-inflammatory cytokine production and pathogen response; while type M2 are associated with anti-inflammatory and homeostatic functions (Zhang and Wang, 2014; Sica et al., 2015). Other groups have found that the excessive inflammation triggered by sepsis can lead to M1 macrophages apoptosis or polarization toward M2 type (Krausgruber et al., 2011; Sindrilaru et al., 2011). As well, some cytokines as TNF-α, IL-4, IL-13, and IL-10, can induce polarization of M1 macrophages to type M2 (Sica et al., 2015; Ip et al., 2017). Similar to these groups, we observed an small increase in the levels of CD86 in M2 cells at both inoculation times; and we found daily differences in the expression levels of CD86 which were slightly higher after inoculation at ZT11 than after ZT19.

Regarding anti-inflammatory mechanisms, we observed that the serum levels of the anti-inflammatory cytokine IL-10 increased in mice injected at both times of LPS administration. Indeed, mice deficient for IL-10 have a higher mortality rate, along with an increase in glucocorticoid and pro-inflammatory cytokines levels (Córdoba-Moreno et al., 2018). Additionally, we analyzed corticosterone levels in serum, which can attain immunosuppressant functions (Stahn and Buttgereit, 2008; Ayroldi et al., 2012), and depend on hypothalamo-pituitary-adrenal (HPA) axis activation. Glucocorticoids were reported to be increased in the serum both of septic patients and animal models (Kwon et al., 2007; Thompson and van Eldik, 2009; Yamashita et al., 2017). In this work, we observed an induction of corticosterone in serum in response to LPS, independently of the inoculation time, suggesting that stimulation of the HPA axis overrules circadian modulation. It was reported a circadian rhythm in the levels of glucocorticoid receptors (Xu et al., 1991) which can be related to the diurnal response to LPS. Therefore, the levels of IL-10 in serum are in line with the levels of corticosterone and of CD86 in type M2 macrophages. These results indicate that the differential daily response is not determined by these anti-inflammatory molecules.

In conclusion, our current results reinforce the idea of the importance of a synchronized circadian clock for septic shock survival and the importance of TNF-α signaling in the daily response to sepsis. The results also evidence a complex communication between the central nervous system and the peripheral tissues, with many signaling pathways working together, which can be responsible for the daily differences observed in response to high doses of LPS. This should be taken into account since patients in the intensive care unit can experience circadian disruption due to artificial lights, medication, mechanical ventilation, pain, among others (Knauert et al., 2015; Boyko et al., 2017).

## Data Availability Statement

The datasets generated for this study are available on request to the corresponding author.

## Ethics Statement

The protocol was approved by the Institutional Animal Care and Use Committee of the National University of Quilmes.

## Author Contributions

MM, IA, CC, LM, and NP performed experiments for the paper. MM and NP analyzed data. MM, NP, and DG wrote the manuscript. DG, LM, and NP provided reagents and funding for the study.

### Conflict of Interest

The authors declare that the research was conducted in the absence of any commercial or financial relationships that could be construed as a potential conflict of interest.
