# Supplementary material for: Differential Thermoregulatory and Inflammatory Patterns in the Circadian Response to LPS-Induced Septic Shock
Source: Front Cell Infect Microbiol. 2020 Mar 12;10:100. doi: 10.3389/fcimb.2020.00100 (PMC7080817; doi:10.3389/fcimb.2020.00100)
Supplement: Supplementary file 1 [file Data_Sheet_1.PDF]

Supplementary Figures

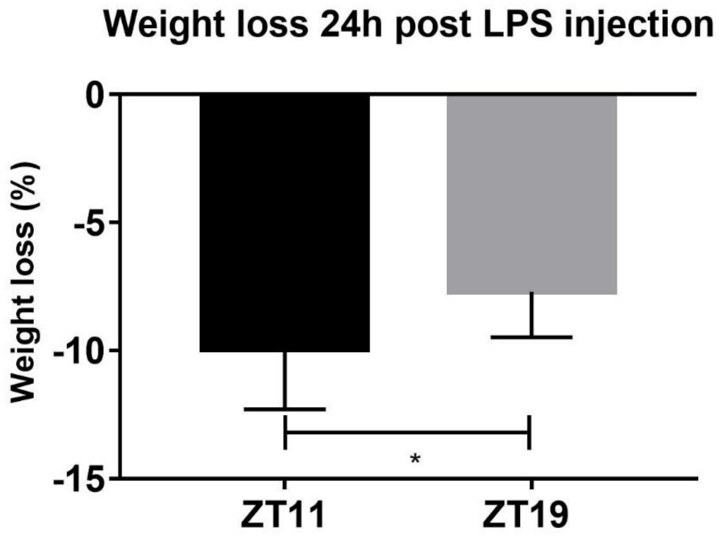

Supplementary Figure 1. Weight loss 24 hours after LPS treatment. Mean ± SEM of the percentage of weight loss 24 hours after LPS treatment at ZT11 or ZT19. Unpaired T-test: *p=0.0248; n=10 per group.

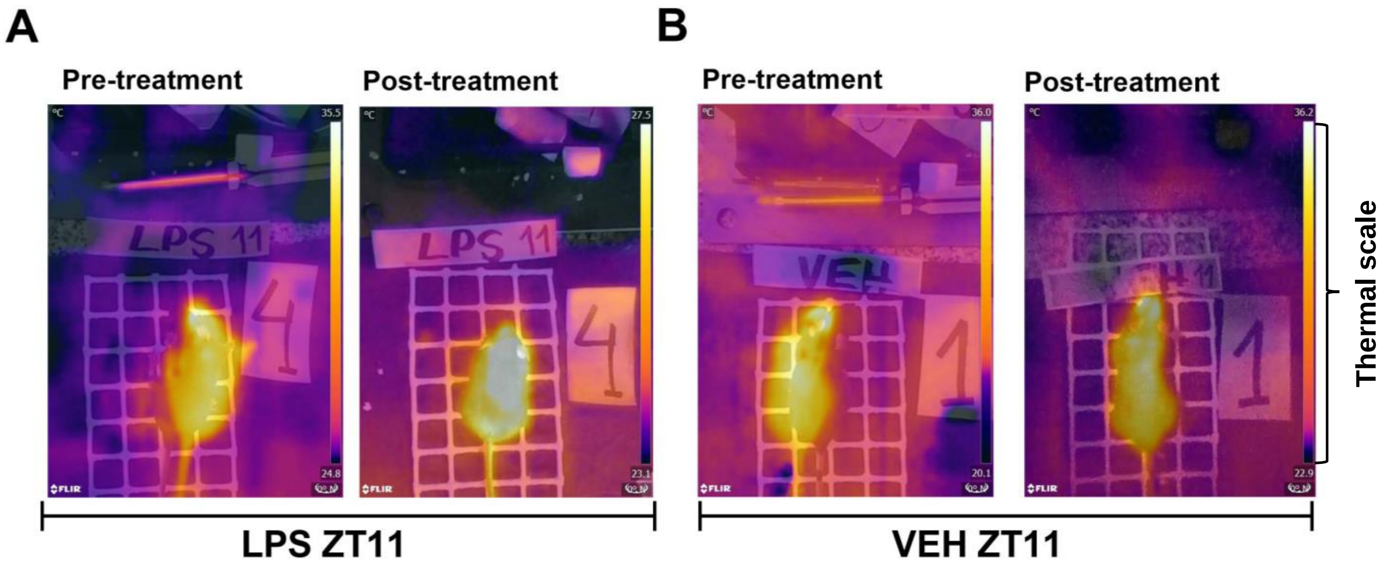

Supplementary Figure 2. Representative thermal pictures obtained with FlirOne® Camera. Pictures taken before and after the treatment of WT mice with LPS (A) or VEH (B) at ZT11. Each colour represents a different temperature within the thermal scale, which is specific for each picture and can be observed at the right of the image. The maximum and minimum temperature of each picture are shown at the ends of the scale. Warm colours represent higher temperatures while cold colours represent lower temperatures.

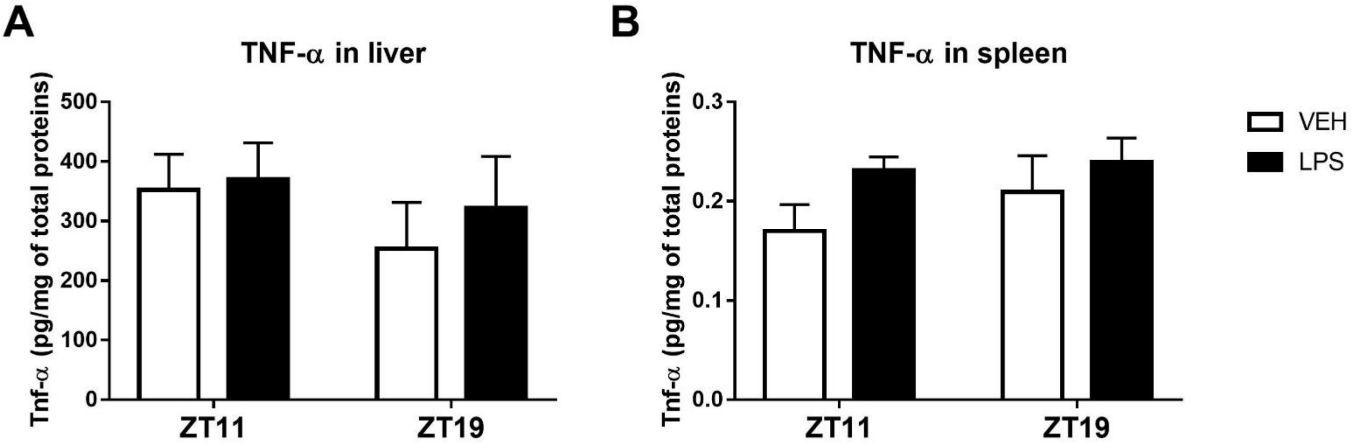

Supplementary Figure 3. TNF-α levels in liver and spleen. Mean ± SEM of TNF-α protein levels **A)** in the liver and **B)** spleen of animals inoculated with 20 mg/kg of LPS or VEH at ZT11 or ZT19. A: n=5 for ZT11 groups and n= 4 for ZT19 groups. B: n=6 per group.

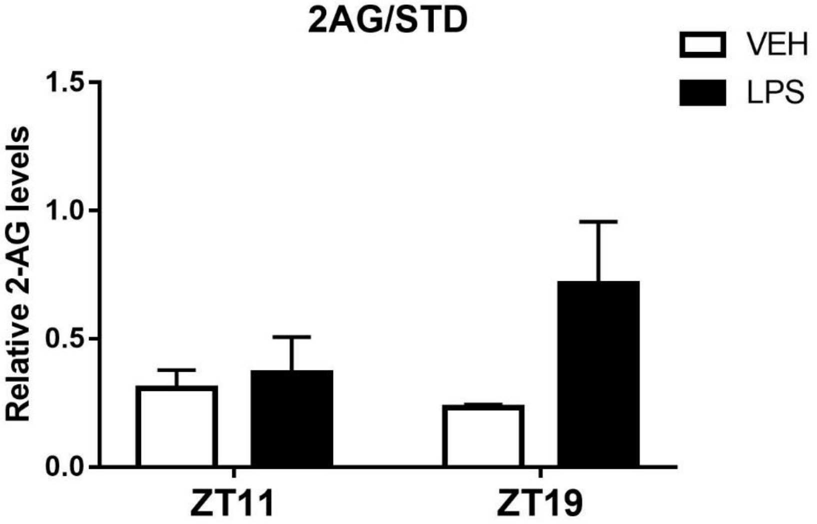

Supplementary Figure 4. Endocannabinoid levels in serum. Mean ± SEM of 2-AG levels in the serum of mice inoculated with 20 mg/kg of LPS or VEH at ZT11 or ZT19. n=4 per group.

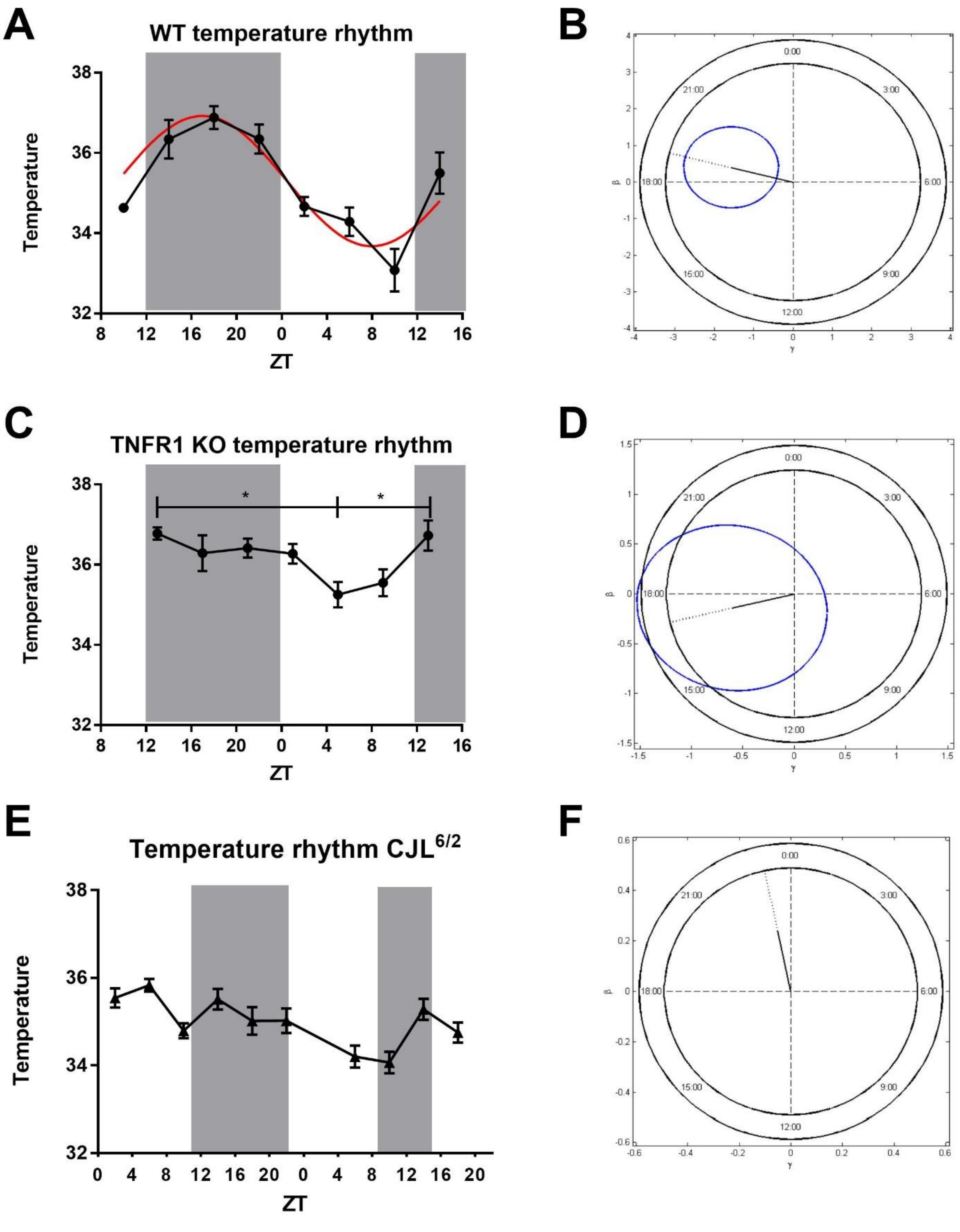

Supplementary Figure 5. Body temperature rhythms. Body temperature monitoring of **A)** WT mice in LD conditions, **C)** TNFR1 KO mice in LD conditions and **E)** WT mice in CJL conditions, recorded using FlirOne® Camera. Cosinor analysis of body temperature variation in **B)** WT animals in LD conditions, **D)** TNFR1 KO mice in LD conditions and **F)** WT mice in CJL conditions. One-way ANOVA: A) $p<0.0001$; C) $p=0.0078$, followed by post-test: *$p=0.0255$ ZT13 (1° day) vs ZT5, *$p=0.0350$ ZT5 vs ZT13 (2° day). B) Cosinor test: $p=0.003$. A-B: n=5 per time point; C-F: n=6 per time point.

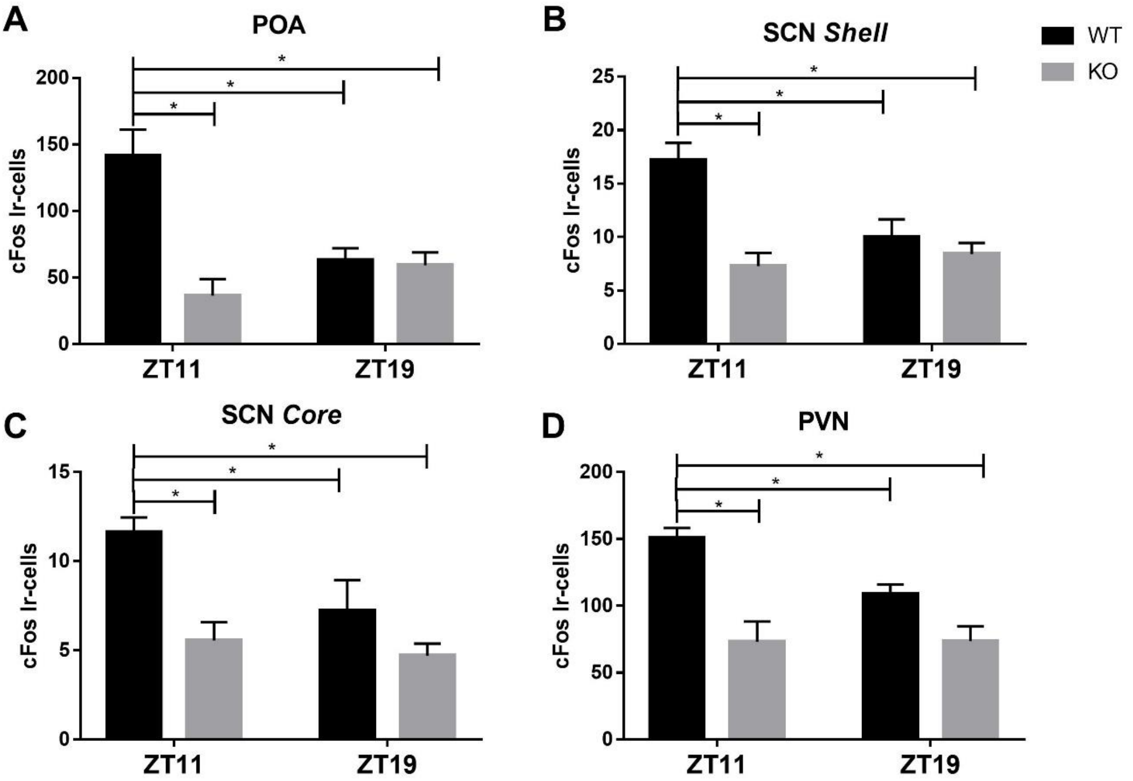

Supplementary Figure 6. Central nervous system activation following LPS treatment in TNFR1 KO mice. Mean ± SEM of the number of cFos immunoreactive (Ir) cells in **A)** POA, **B)** shell and **C)** core of the SCN and **D)** PVN of TNFR1 KO and WT mice inoculated with 20 mg/kg of LPS at ZT11 or ZT19. Kruskall Wallis test: A) p=0.0025, B) p=0.0061, C) p=0.0094 and D) p=0.0002; followed by post-test: *p<0.05 for WT ZT11 vs all the other groups in all the regions studied. n= 10 for WT ZT11, n= 11 for WT ZT19, n=5 for KO ZT11 and n=5 for KO ZT19; except for SCN regions n=9 for WT ZT11, and for PVN n=4 for KO ZT11.

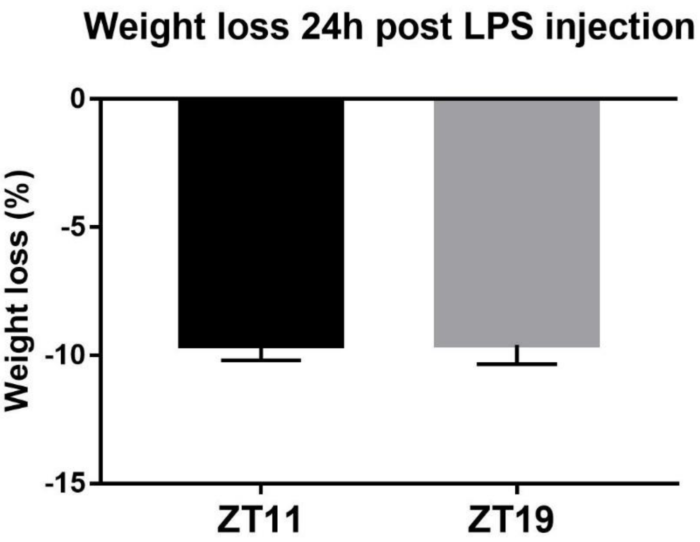

Supplementary Figure 7. Weight loss 24 hours after LPS treatment in CJL$^{6/2}$ conditions. Mean ± SEM of the percentage of weight loss 24 hours after LPS treatment at ZT11 or ZT19 in animals subjected to CJL$^{6/2}$ conditions. n=10 per group.
